# The place of physical activity in the WHO Global Strategy on Diet and Physical Activity

**DOI:** 10.1186/1479-5868-2-10

**Published:** 2005-08-24

**Authors:** Adrian Bauman, Cora L Craig

**Affiliations:** 1 Center for Physical Activity and Health, School of Public Health, University of Sydney, Sydney Australia 2006; 2 Canadian Fitness and Lifestyle Research Institute, Ottawa Canada

## Abstract

In an effort to reduce the global burden of non-communicable disease, the World Health Organization released a Global Strategy for Diet and Physical Activity in May 2004. This commentary reports on the development of the strategy and its importance specifically for physical activity-related work of NGOs and researchers interested in increasing global physical activity participation.

Sparked by its work on global efforts to target non-communicable disease prevention in 2000, the World Health Organization commissioned a global strategy on diet and physical activity. The physical activity interest followed efforts that had led to the initial global "Move for Health Day" in 2002. WHO assembled a reference group for the global strategy, and a regional consultation process with countries was undertaken. Underpinning the responses was the need for more physical activity advocacy; partnerships outside of health including urban planning; development of national activity guidelines; and monitoring of the implementation of the strategy.

The consultation process was an important mechanism to confirm the importance and elevate the profile of physical activity within the global strategy. It is suggested that separate implementation strategies for diet and physical activity may be needed to work with partner agencies in disparate sectors (e.g. urban planning for physical activity, agriculture for diet). International professional societies are well situated to make an important contribution to global public health by advocating for the importance of physical activity among risk factors; developing international measures of physical activity and global impacts of inactivity; and developing a global research and intervention agenda.

## Introduction

Physical inactivity is recognized as a major risk factor for non-communicable diseases (NCDs), and ranks between the second and sixth most important risk factor in contributing to the population burden of disease in westernized countries [1-3]. The increasing global problem of NCDs means that obesity, poor diet and inactivity are increasing problems for countries in the epidemiological transition [4].

From a physical activity standpoint, it is interesting to reflect on the temporal relationship between the accrual of evidence and the time delays to the development of policy frameworks for action. Initial epidemiological studies in the 1950s and 1960s identified, for the first time, population level evidence that inactivity was a risk factor for cardiovascular disease or for all cause mortality [5,6]. This evidence continued to accumulate, such that by 1987 a systematic review reported a consistent relationship between inactivity and cardiovascular disease [7]. Three years later, this was confirmed by a formal meta-analysis [8]. This period and the following half dozen years was characterized by increased interest and advocacy by physical activity researchers and organizations, resulting in consensus statements and a US Surgeon General's Report [9,10]. Gradually, some countries engaged with the physical activity agenda, developed guidelines and started to identify physical activity related health targets [10-12]. However, most countries paid little attention to addressing levels of inactivity in a systematic manner.

Over the past few years the World Health Organization (WHO) has become interested in NCD prevention as a global health concern, fueled by WHO discussion and a resolution to focus on NCD prevention and control in mid 2000 [13]. This document urged countries to "to develop national policy frameworks... to create conducive environment for healthy lifestyles... (largely due to) unhealthy diet, physical inactivity and tobacco use" [13].

Interest in NCD prevention led to reflections on the contributory risk factors, and in a global context, diet and inactivity became issues of concern. Increasing rates of obesity among youth have been recognized since the 1960s [14], and since around 1980 among adults [15], but quite suddenly since the late 1990s, obesity has received increasing political and media interest. This further contributed to increased international interest in inactivity and poor diet by around 2000 or 2001. Furthermore, following the advocacy and efforts stimulated by the Agita programs in South America created interest in developing countries [16]. As a consequence of these efforts, and of the WHA53.17 resolution, the Director General of WHO in 2001 recommended that world Health Day in 2002 should be physical activity focused, and the 'Move for Health' initiative was launched in early 2002 [17].

This commentary reports on the international development of the 2004 WHO Global Strategy for Diet and Physical activity, viewed from the physical activity perspective. The purposes are to report on the development of the strategy, to show how physical activity was positioned during and after the strategy was developed, and to indicate the potential importance of the strategy for the physical activity related work of international organizations, professional societies and researchers interested in the physical inactivity as a global public health problem.

## Discussion of the Global strategy – through development to implementation

### Physical activity and the development of the Global Strategy

WHO recognized the need and commissioned a global strategy on diet and physical activity at its 56^th ^World Health Assembly [18]. An important influencer was the earlier #916 report by WHO/FAO [19] which indicated the health risks of obesity and overnutrition, and their contribution to global ill-health. The #916 report also indicated the benefits of physical activity on cardiovascular disease, diabetes and for osteoporosis prevention and mentioned the IARC report on the cancer prevention role of physical activity and weight control [20] which had influenced its development. Physical activity was included as an adjunctive idea, obviously contributing to the energy expenditure side of energy balance, but was not an initial impetus for the strategy.

One important contribution to physical activity was the development of the WHO 'Move for Health' day. This had emanated out of the local and national work of Agita!, a community-wide physical activity and advocacy program which started in the 1990s in the San Caetano region of Sao Paulo [16,17]. This initiative started in Brazil, but spread to other parts of South America, and finally led to WHO interest, and to World Health Day in April 2002. Since then, annual 'Move for Health' day work has occurred under the auspices of WHO, as well related efforts through the Agita Mundo NGO in Brazil [21].

Thus the WHO strategy development had a mandate to consider both 'diet and physical activity', and an Expert Reference Group was convened in 2002. This 14 member group primarily consisted of nutrition-oriented experts, but two [the authors of this commentary] had specific physical activity expertise.

The process of developing the strategy comprised several stages. A draft strategy was written by WHO staff and the Expert Reference group by late 2002, and then processes of consulting with individual countries (and through WHO regional consultations), the private sector, NGOs and other UN agencies occurred [22,23].

**Figure 1:**
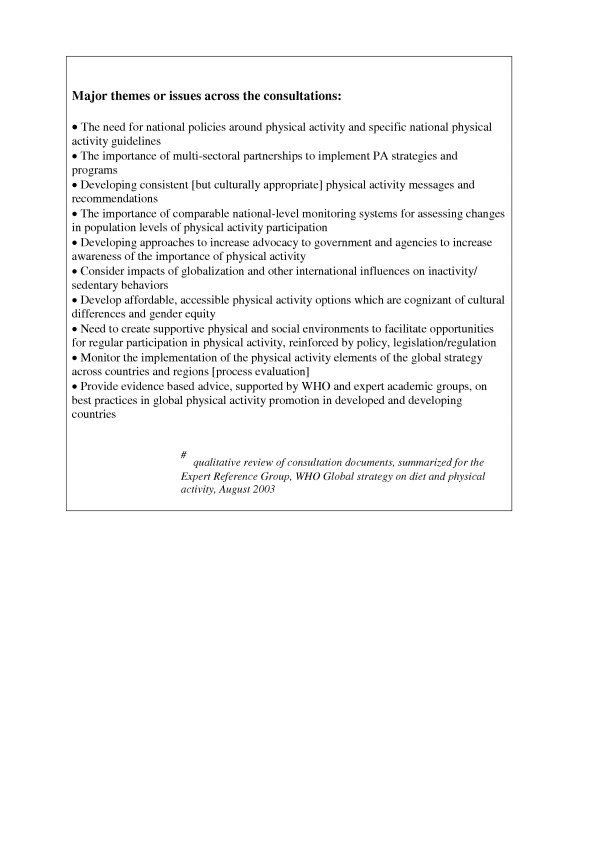
How the world characterized the most important physical activity issues relevant to the development of a global strategy, 2003 #.

These discussions and consultations considered diet and physical activity. The feedback from most regions reflected roughly equivalent concern with issues related to diet and physical activity; only one region focused solely on diet related issues. The authors prepared a summary of the physical activity-specific themes emanating from the regional discussions and consultations and submitted this distillation to the Expert Reference group in August 2003 [24]. This was a qualitative review across the regional and country consultations, public web forum, NGO and private sector reports, and UN agencies consultations. It describes 'how the world viewed the important physical activity issues in 2003', from the perspective of the global strategy. The main themes are shown in Figure 1 [adapted from [24]].

Underpinning all the responses from the consultations was the need to advocate more widely to governments for coordinated planning and resources for physical activity. In addition, there was widespread recognition that population-wide physical activity efforts need interagency collaboration and partnerships; physical activity promoting efforts require substantial work with other effector agencies outside of health, including departments of transport, urban planning, education and sport, most of which differ from those for nutrition promotion. This is consistent with previous WHO frameworks, dating back to the Ottawa Charter [25]. This intersectoral work will allow the development of health enhancing physical environments, and the development of multi-level policies that could support physical activity efforts [26]. One corollary of this is that, if the effector arms of physical activity programs are different to nutrition, then strategic implementation and partnerships might be kept separate, although the health-related consequences of poor diet and physical inactivity show large degrees of overlap.

Although some countries have physical activity guidelines, these tend to be in developed nations [10-12]. The extent of physical activity policies is even less well documented, and sometimes these are embedded in other policy documents for NCD prevention or obesity [27]. WHO was seen as responsible for supporting guideline development, as well as providing technical support for the evidence base for intervention, particularly in developing countries.

The importance of increasing community awareness was highlighted, but in order to achieve this, consistent physical activity messages are required. The recommendation of 'half an hour of achievable moderate intensity activity on most days of the week' [10] fits well into social marketing and media campaign efforts. Additional physical activity, or activity at a greater intensity may be required for some health outcomes such as cancer prevention or weight loss, but making the message(s) more complicated may confuse efforts to raise community awareness.

Finally, monitoring the implementation of the Strategy was thought to be important – documenting what happens by country, region and at the NGO level would provide a useful framework for assessing the actions undertaken relevant to the Global strategy. This process evaluation should be supplemented by the development of national monitoring systems, to assess and compare epidemiological trends in physical activity behaviors over time.

Physical activity was given emphasis in some regional consultations, especially the WHO Western Pacific region, Europe, and the Pan-American region. The latter region, armed with an already existing physical activity network (RAFA) [17], focused on the need for paradigm shifts, from an emphasis on sport to a new focus on 'active living' [28].

### The launch of the Global Strategy and its sequelae

The development of the Global Strategy generated much political interest, media attention and controversy especially around nutrition. Concerns expressed by some Governments and by the private sector influenced the levels of agreement with, and content of the Strategy [29]. Changes made during the preparation of the Global Strategy were mostly confined to nutrition, rather than physical activity [30]. Nutrition appeared to be more controversial, but was sometimes given more emphasis as the "most important" risk factor by some writers [30].

The physical activity elements in the Global Strategy were mostly unaltered by the politics of consultation and revision. One general reference to 'individual responsibility' for physical activity and health was attenuated in the final version by framing individual choices in the context of health promoting environments [18]. The Global Strategy was approved by the World Health Assembly of WHO in May 2004 [18]. It provided a platform for advocacy, and an international 'call to action' to reduce NCD risk factors.

After its release, the Global Strategy received ongoing media attention. The media tended to be overly focused on the nutrition controversy, and sometimes even incorrectly described it as a global "obesity strategy". However, sometimes commercial interests became suddenly interested in funding physical activity promotion programs, perhaps to orient decision-makers and political attention away from the 'overheated' nutrition debate. Thus, large scale partnerships with the private sector around physical activity need to be considered carefully and ethically before rushing into conjoint program development.

## Conclusion

The regional consultations were an important mechanism for developing the global strategy. They affirmed the relevance of physical activity in most WHO regions, and added emphasis to physical activity within the overall strategy. The qualitative analysis identified that the regional consultations had played a key advocacy role that helped to drive the physical activity agenda from the periphery, and hence its profile was increased in the final document.

It is suggested that separate implementation of diet and physical activity strategies is needed since there are different effector agencies. Transportation, sport agencies, recreation and urban planning policies are integral to physical activity promotion, whereas agricultural food policy and trade are more central to diet; there is not an automatic overlap of partnerships with these disparate agencies for implementation of the strategy. This is different to a 'health oriented' approach, which sees commonalities and integration only within a non-communicable disease framework, or within approaches to obesity prevention and control. These should be utilized, as the overall objective of the Global Strategy is to reduce NCDs. Nonetheless, engaging with agencies and partnerships outside health is a valuable approach to fostering commitment to developing and resourcing programs.

There clearly needs to be a greater commitment to ongoing population level physical activity measurement. Some efforts at developing global instruments for measuring physical activity have commenced, including the International Physical Activity Questionnaire (IPAQ), which has been shown to be reliable and valid in 12 countries [31]. Other efforts, through the WHO Steps surveillance system, are trialing the Global Physical Activity Questionnaire (GPAQ), a domain-specific short version of the IPAQ instrument [32].

The adoption of the global strategy by the WHO Assembly is a unique opportunity in the history of international physical activity work, as the development of common frameworks, policies and programs would enable greater program opportunities and partnerships at the national level. However, no resources have been earmarked to do this work, and implementation plans remain to be developed. Efforts to engage with countries and move this agenda forward are under way at the regional levels of WHO, with support and advocacy from NGOs and professional groups and societies.

As an international society, ISBNPA has greater potential to contribute to 'big picture participation in global work', compared to national organizations representing obesity or exercise science. The challenges posed by working with the Global strategy are very different to scholarly academic work; this engagement with the Global strategy is unpaid work, requires advocacy with governments and decision makers and is not often rewarded by academic funding or publication. The benefits are in making contributions to real population health efforts, and in improving the underecognised profile of physical activity among risk factors. The International Society of Behavioral Nutrition and Physical Activity (ISBNPA) had supported the development of the Global Strategy through a formal correspondence with WHO; now is a unique time for such organizations and their constituent members to contribute to the great challenges of international population behavior change.

The potential roles of organizations such as ISBNPA, American College of Sport Medicine, International Association for the Study of Obesity and others are in a few key areas of research and policy. First, advocacy for physical activity, to keep it on the political and health agenda of national and regional governments, especially advocating in transitional countries where the burden of NCDs will increase dramatically in the coming decades [34]. Second, it is important to move physical activity to a 'whole of government' agenda, to include a range of agencies, such as sport, transport and urban planning, as well as the private sector and NGOs [27]. Organizations such as ISBNPA can be research and policy brokers in fostering these relationships, and in adopting standard internal policies, which are consistent and could be applied in different contexts. Finally, in terms of science, members of ISBNPA could contribute to the global research and surveillance agenda [33].

The research challenges in the international context are worthy of more urgent efforts. For example, developing international measures of physical activity remains difficult. There are trade offs between capturing several domains of activity [versus one leisure time domain]. Measurement validity is difficult to establish, not only against criterion objective measures of movement or fitness, but in the varied cultural contexts and many different meanings that may be applied to the same self reported physical activity questions in different countries. Other research challenges include the definition of global impacts on inactivity. The globalization research agenda could include studies of trends in occupational physical activity, changes to the domestic and urban environments, factors contributing to the development of pervasive sedentary lifestyles, and monitoring declines in active commuting [35]. The measurement development agenda includes establishing and using standard process and impact indicators to assess the implementation of the global strategy across sectors and populations. Finally, the evaluation designs around a global initiative preclude planned comparison groups. Assessing the impact on physical activity in countries with active policy and resourced programs can be compared to demographically similar countries with limited program development. The international research challenges here are vast, and more complex than controllable smaller sample research in developed countries. Case studies of best practice may be one possible solution, provided the evidence from these is disseminated widely. Nonetheless, for those who are serious about public health approaches to increasing physical activity, global engagement is a necessary component of the work that we have yet to do.

## Competing interests

The author(s) declare that they have no competing interests.

## Authors' contributions

both authors were the Physical Activity representatives on the Expert Reference group, WHO Global Strategy on diet and physical activity 2002–2004; both contributed to the conceptualizing and writing of this paper. The views and opinions expressed in this commentary are solely those of the authors.
